# Eastern Equine Encephalitis in Children, Massachusetts and New Hampshire,USA, 1970–2010

**DOI:** 10.3201/eid1902.120039

**Published:** 2013-02

**Authors:** Michael A. Silverman, John Misasi, Sandra Smole, Henry A. Feldman, Adam B. Cohen, Sandro Santagata, Michael McManus, Asim A. Ahmed

**Affiliations:** Author affiliations: Boston Children's Hospital, Boston, Massachusetts, USA (M.A. Silverman, J. Misasi, H.A. Feldman, S. Santagata, M. McManus, A.A. Ahmed);; Department of Public Health, Boston (S. Smole); Massachusetts General Hospital, Boston (A.B. Cohen);; Brigham and Women’s Hospital, Boston (S. Santagata)

**Keywords:** Encephalitis, arbovirus, Eastern equine encephalitis, Eastern equine encephalitis virus, pediatric, Massachusetts, New Hampshire, children, viruses

## Abstract

A short prodrome in children is associated with more severe disease and increased risk for death.

## Medscape CME Activity

Medscape, LLC is pleased to provide online continuing medical education (CME) for this journal article, allowing clinicians the opportunity to earn CME credit. 

This activity has been planned and implemented in accordance with the Essential Areas and policies of the Accreditation Council for Continuing Medical Education through the joint sponsorship of Medscape, LLC and Emerging Infectious Diseases. Medscape, LLC is accredited by the ACCME to provide continuing medical education for physicians.

Medscape, LLC designates this Journal-based CME activity for a maximum of 1 *AMA PRA Category 1 Credit(s)*^TM^. Physicians should claim only the credit commensurate with the extent of their participation in the activity.

All other clinicians completing this activity will be issued a certificate of participation. To participate in this journal CME activity: (1) review the learning objectives and author disclosures; (2) study the education content; (3) take the post-test with a 70% minimum passing score and complete the evaluation at www.medscape.org/journal/eid; (4) view/print certificate.

**Release date: January 16, 2013; Expiration date: January 16, 2014**

## Learning Objectives

Upon completion of this activity, participants will be able to:

- Assess the clinical presentation of eastern equine encephalitis (EEE).
- Distinguish common ancillary findings in cases of EEE.
- Analyze risk factors for poor outcomes of EEE.
- Evaluate the most common outcomes of EEE.

## CME Editor

**Claudia Chesley, **Technical Writer/Editor, Emerging Infectious *Diseases. Disclosure: Claudia Chesley has disclosed no relevant financial relationships.*

## CME Author

**Charles P. Vega, MD, **Health Sciences Clinical Professor; Residency Director, Department of Family Medicine, University of California, Irvine. *Disclosure: Charles P. Vega, MD, has disclosed no relevant financial relationships.*

## Authors

*Disclosures:* ***Michael A. Silverman, MD*** *, owns stock, stock options, or bonds from Pfizer.* ***John Misasi, MD*** *;* ***Sandra Smole, PhD*** *;* ***Henry A. Feldman, PhD*** *;* ***Adam B. Cohen, MD*** *;* ***Sandro Santagata, MD, PhD*** *;* ***Michael McManus, MD*** *; and* ***Asim A. Ahmed, MD*** *, have disclosed no relevant financial relationships.*
