# Supplementary material for: Eastern Equine Encephalitis in Children, Massachusetts and New Hampshire,USA, 1970–2010
Source: Emerg Infect Dis. 2013 Feb;19(2):194–201. doi: 10.3201/eid1902.120039 (PMC3559032; doi:10.3201/eid1902.120039)
Supplement: Related texts

# Review article: Eastern Equine Encephalitis in Children, Massachusetts and New Hampshire,USA, 1970–2010

**Authors:** Michael A. Silverman, John Misasi, Sandra Smole, Henry A. Feldman, Adam B. Cohen, Sandro Santagata, Michael McManus, Asim A. Ahmed

**Affiliations:** Author affiliations: Boston Children's Hospital, Boston, Massachusetts, USA (M.A. Silverman, J. Misasi, H.A. Feldman, S. Santagata, M. McManus, A.A. Ahmed);; Department of Public Health, Boston (S. Smole); Massachusetts General Hospital, Boston (A.B. Cohen);; Brigham and Women’s Hospital, Boston (S. Santagata)

## Abstract

We describe the clinical, laboratory, and radiographic characteristics of 15 cases of eastern equine encephalitis in children during 1970–2010. The most common clinical and laboratory features were fever, headache, seizures, peripheral leukocytosis, and cerebrospinal fluid neutrophilic pleocytosis. Radiographic lesions were found in the basal ganglia, thalami, and cerebral cortex. Clinical outcomes included severe neurologic deficits in 5 (33%) patients, death of 4 (27%), full recovery of 4 (27%), and mild neurologic deficits in 2 (13%). We identify an association between a short prodrome and an increased risk for death or for severe disease.

Eastern equine encephalitis (EEE) is a highly virulent reemerging arboviral encephalitis in humans; the disease is endemic to the eastern United States and the Gulf Coast (1,*2*). EEE is characterized by a nonspecific prodrome followed by severe headache, high fevers, lethargy, and seizures (1,3,4). The meningoencephalitis often progresses rapidly to coma and death; mortality rates are 30%–70% (4). On average, there are 6 cases per year in the United States, some occurring sporadically and others in epidemics (5). Over the past decade, EEE virus (EEEV) transmission has resurged in the northeastern region of North America. In the United States, overlapping epidemics among humans occurred in Massachusetts during 2004–2006 and in New Hampshire during 2005 and 2007 (1). During consecutive years of EEEV activity from 2009 to 2011, New York State reported its first human cases of EEE in 26 years (6). In 2012, a total of 7 human cases of EEE were diagnosed in Massachusetts (7)—this is the highest number of annual cases in the state since 1956. Moreover, the first human cases of EEE in Vermont were reported in 2012 (8), and public health surveillance indicates that the range of EEEV has extended into the state of Maine (9) and into Nova Scotia, Canada (10).

The natural history of EEEV infections in children has not been well characterized. This mosquito-borne illness in humans was first described during an outbreak in Massachusetts in 1938 (11), and Farber et al. (12) described 8 cases of EEE in children during that outbreak. Since then, case series of EEE have been published (1,3,4,13), but investigations of EEE in children are lacking. We conducted this study to determine the clinical, laboratory, radiographic, and pathologic features of EEE in children and to assess features associated with the clinical outcome of EEE in children.

## Methods

### Data Sources

Cases of EEE at Boston Children’s Hospital in Boston, Massachusetts, during 1970–2010 were identified by searching hospital records and records of the Massachusetts Department of Public Health. Institutional review board approval was obtained.

### Case Definitions and Laboratory Confirmation

Case-patients were defined as children with illness compatible with encephalitis and with laboratory confirmation of EEEV infection (14). EEE was confirmed by the William A. Hinton State Laboratory Institute, Department of Public Health, Boston, on the basis of at least 1 of the following: isolation of EEEV or demonstration of specific viral antigen or genomic sequence from cerebrospinal fluid (CSF) or brain tissue; detection of virus-specific IgM in CSF by antibody-capture ELISA; >4-fold increase in acute-phase versus convalescent-phase serum antibodies; or detection of EEEV-specific IgM in CSF by ELISA, followed by confirmation of virus-specific neutralizing antibodies by plaque-reduction neutralization assay.

### Case-Patient Information

A records search revealed a total of 15 cases; medical records were available for 14. Partial records from the Massachusetts Department of Public Health were available for patient 2. Patient 1 has been described (15). We defined the illness prodrome as the time between initial nonspecific symptoms and the first major neurologic symptom, such as headache with photophobia, stiff neck, altered mental status, or seizures. We used the Pediatric Cerebral Performance Category Scale (PCPC) to classify outcomes, which were determined on the basis of the clinical condition of the patient at the time of hospital discharge (16). Normal, mild, and moderate disabilities were classified as favorable outcomes (PCPC scores 1–3); severe disability, coma, and death were classified as unfavorable outcomes (PCPC scores 4–6).

### Radiograph Interpretation

Radiographs were reviewed by a neuroradiologist who was aware of the EEE diagnosis but blinded to the specifics of the patient’s history and previous radiographic and pathologic findings. Lesions seen on radiographs were scored on a scale of 0–3 for the degree of T2 fluid-attenuated inversion recovery enhancement (0, no lesion; 1, 2, and 3, minimal, moderate, and severe enhancement, respectively).

### Pathology

Postmortem CNS specimens from 2 patients were analyzed by histopathologic and immunohistochemical examination. Control CNS tissues were postmortem specimens from patients with other forms of encephalitis.

### Statistical Methods

The Mann-Whitney rank-sum test was used to compare the length of prodromes for patients with favorable outcomes with those for patients with unfavorable outcomes; p<0.05 was considered statistically significant. Pearson’s correlation was used to determine relationships between continuous variables.

## Results

### Demographic and Epidemiologic Characteristics

During 1970–2010, a total of 19 cases of EEE in children in Massachusetts and New Hampshire were reported to the Centers for Disease Control and Prevention; our case series includes 15 of those 19 cases. Thirteen of the patients were from Massachusetts and 2 were from New Hampshire. The median age was 5.3 years (range 0.5–14.7 years). Medical care was sought for all patients during August, September, or October. The clinical features, diagnostic results, treatments, and outcomes for each patient are described in Table 1.

**Table 1:** Clinical features of and diagnostic studies for 15 children with eastern equine encephalitis, Massachusetts and New Hampshire, 1970–2010*

Patient no.	Admitted to hospital	Age, y/sex	Prodome length, d	Blood leukocytes, 10^3^ cells/μL	CSF leukocytes, cells/μL	CSF protein, mg/dL	ICP checked	Treatment	Outcome at discharge	Status at follow-up
1	1970 Aug	0.5/M	2	28.0	300	64	No	AEDs, mannitol	Severe disability	No change
2	1974 Aug	1.2/M	2	NA	NA	NA	NA	NA	Death	–
3	1982 Sep	0.8/M	1	17.1	11	66	No	Vidarabine, acyclovir, AEDs, dexamethasone	Severe disability	Improved
4	1982 Sep	14.7/F	2	17.3	1,004	80	Yes	AEDs, mannitol	Severe disability	Improved
5	1983 Aug	11.2/M	3	21.0	720	61	No	Mannitol	Full recovery	No change
6	1984 Aug	6.5/F	0	21.0	1,384	46	Yes	Acyclovir, AEDs, pentobarbitol coma, dexamethasone, mannitol	Severe disability	Improved
7	1995 Aug	0.5/M	4	13.6	451	73	No	Acyclovir, AEDs, IVIG, dexamethasone, cyclophosphamide	Severe disability	NA
8	2000 Sep	4.0/F	5	22.7	258	33	No	Acylovir, AEDs	Full recovery	No change
9	2001 Aug	3.9/F	5	12.0	1,200	54	No	Acylovir, AEDs	Mild to moderate disability	Improved
10	2004 Aug	13.1/M	0	8.4	860	84	No	Acylovir, AEDs dexamethasone, mannitol, 3% saline	Death	–
11	2004 Oct	11.5/F	8	16.5	616	73	No	Acylovir	Full recovery	No change
12	2005 Aug	5.3/F	2	34.8	847	73	No	Acylovir, AEDs, 3% saline	Death	–
13	2006 Aug	9.9/M	2	23.0	1,085	81	Yes	Acylovir, AEDs, pentobarbitol coma, interferon α-2b, dexamethasone, IVIG, 3% saline	Death	–
14	2007 Aug	13.3/M	6	20.4	888	124	No	Acylovir, AEDs, IVIG, 3% saline	Mild to moderate disability	No change
15	2009 Sep	3.5/F	5	17.8	258	39	No	Acylovir, AEDs, dexamethasone	Full recovery	No change

*CSF, cerebrospinal fluid; ICP, intracranial pressure; AEDs, anti-epileptic drugs (phenobarbital, phenytoin, fosphenytoin, carbamazepine, oxcarbazepine, lorazepam, levetiracetam); ND, not done; –, patient died or did not return for follow-up; IVIG, intravenous immunoglobulin.

### Clinical and Laboratory Characteristics

Of the 15 patients, 4 (27%) died, 5 (33%) had severe neurologic sequelae, 2 (13%) had mild to moderate deficits, and 4 (27%) fully recovered. The median duration of hospitalization was 16.0 days. The median duration of hospitalization for patients who died was 6.6 days.

Fever was reported for all patients on or near day 1 of hospitalization. Signs and symptoms of meningoencephalitis, such as seizures, headaches, nausea, vomiting, and stiff neck, were observed for all patients (Table 2). Of the 10 patients who had seizures, 5 had complex partial seizures, 3 had generalized tonic–clonic seizures, and 2 had both types of seizures.

**Table 2:** Clinical signs and symptoms at the time of hospital admission for 14 children with eastern equine encephalitis, Massachusetts and New Hampshire, 1970–2010

Sign or symptom	No. (%) patients
Fever	14 (100)
Seizure	10 (71)
Headache	9 (64)
Neck stiffness	9 (64)
Nausea/vomiting	9 (64)
Myalgia/arthralgia	4 (29)
Plantar reflex	4 (29)
Photophobia	4 (29)
Sore throat	2 (14)
Cranial nerve palsy	1 (7)
Abdominal pain	1 (7)
Diarrhea	0

The prodrome phase lasted >3 days for 7 patients and <3 days for 8 patients (median 2 days, range 0–8 days) (Table 1). Patients with a longer prodrome typically reported a few days of nonspecific symptoms, such as fever, headache, and nausea followed by slower progression of neurologic symptoms, including photophobia, stiff neck, altered mental status, and seizures. In contrast, patients with a shorter prodrome often had rapidly declining mental status and seizures. Of the 15 patients, 13 (87%) became stuporous or comatose during the first 3 days of hospitalization (median 2 days). The remaining 2 patients became delirious but remained responsive.

CSF samples from all patients had neutrophilic pleocytosis, and 12 (86%) had elevated levels of protein (Table 3). Lumbar puncture was performed a median of 2.0 days (range 0–8 days) after symptom onset. Of 14 patients, 13 (93%) had an elevated peripheral leukocyte count on or before day 1 of hospitalization, and 8 (57%) had peripheral neutrophilia.

**Table 3:** Laboratory values at the time of hospital admission for 14 children with eastern equine encephalitis, Massachusetts and New Hampshire, 1970–2010

Variable	Median value (range)	Reference range
Blood		
Leukocytes, thousand cells/μL	19.1 (8.4–34.8)	5.7–9.9
Neutrophils, %	84 (40–95)	39–77
Hematocrit, %	36.5 (30–43.9)	31.5–38.0
Platelets, thousand cells/μL	230 (155–416)	198–371
Sodium mmol/L	137 (130–144)	135–148
Cerebrospinal fluid		
Leukocytes, cells/μL	784 (11–1,384)	0–5
Neutrophils, %	83 (61–100)	0–2
Red blood cells, cells/μL	10 (0–721)	0
Glucose, mg/dL	78 (40–135)	60–80
Total protein, mg/dL	70 (33–124)	15–45

Electroencephalogram results were available for 14 patients. A pattern of generalized slowing of brain electrical activity was observed in 13 patients; subclinical status epilepticus was noted for 1 of these patients, and epileptiform activity was noted for 5. No correlation could be drawn between electroencephalogram findings and outcome.

### Neuroimaging Findings

Six of the 9 initial head computed tomography (CT) scans were abnormal. The initial CT scan was performed at a mean of 1 day (range 0–2 days) after the onset of neurologic symptoms. Lesions were most commonly found in the basal ganglia, cerebral cortex, and thalamus (Table 4 and Figure 1, panel A). These abnormalities were often subtle and initially were underreported, particularly the cortical lesions.

**Table 4:** Results of neuroimaging studies for 9 children with eastern equine encephalitis, Massachusetts and New Hampshire, 1970–2010*

Location studied	No. (%) patients with abnormal finding	
	MRI, n = 8	CT, n = 9
Meninges	8 (100)	0
Cortex	7 (88)	5 (56)
Basal ganglia	7 (88)	6 (67)
Thalamus	5 (63)	4 (44)
Brainstem	2 (25)	2 (22)
Subcortical white matter	1 (13)	1 (11)
Periventricular white matter	0	1 (11)
Hydrocephalus	0	2 (22)
Any	8 (100)	6 (67)

*MRI, magnetic resonance imaging; CT, computed tomography.

**Figure 1:** Magnetic resonance images (MRIs) and computed tomography (CT) neuroradiographs showing lesions in brains of 3 children with eastern equine encephalitis A) Results of noncontrast CT scan of the brain of patient 12 on hospital day 2; the neuroradiograph shows subtle hypoattenuation of the left caudate head (arrow) and diencephalic region. B) Axial fluid attenuated inversion recovery (FLAIR) image from brain MRI scan of patient 14 on hospital day 2; the image shows abnormal T2 hyperintense regions of the bimesial temporal regions (thick arrows) with accompanying abnormal T2 hyperintense regions of the dorsal pontomesencephalic regions (thin arrows). C, D) FLAIR images from brain MRI scan of patient 15 on hospital day 3. C) Abnormal T2 hyperintense caudate and thalamic nuclei, most prominent on the right (arrow). D) Abnormal T2 hyperintense regions are most prominent in the right parietotemporal gray matter (arrow) and subcortical white matter but are also seen scattered throughout.

Magnetic resonance imaging (MRI) was performed for 12 patients; all images showed evidence of abnormalities. The initial MRI was performed a mean of 2 days (range 1–4 days) after onset of neurologic symptoms. MRI results most commonly revealed abnormalities in the meninges, the cerebral cortex, basal ganglia, and thalamus (Table 4 and Figure 1, panels B–D). Lesions were most apparent on fluid-attenuated inversion recovery, diffusion-weighted imaging, and T1-weighted postgadolinium sequences, and they were more evident on MRI than CT images. The abnormalities seen on MRI or CT colocalized with areas of inflammation and EEE virus seen in pathology specimens (Table 5), but the location and intensity of the abnormalities seen on images did not correlate with clinical outcomes.

**Table 5:** Colocalization of lesions, virus, and brain inflammation in 2 children in a study of EEE, Massachusetts and New Hampshire, 1970–2010*

Patient, anatomical location	Inflammation†	EEE virus†	MRI showing lesion(s)‡
Patient 10			
Frontal cortex	+++	+++	+++
Parietal cortex	++	++	++
Temporal cortex	++	+++	++
Occipital cortex	+	–	–
Thalamus	+++	+++	++
Basal ganglia	++	+++	+++
Cerebellar cortex	+	+	–
Patient 12			
Frontal cortex	++	++	++
Parietal cortex	+	+	–
Temporal cortex	++	++	–
Occipital cortex	+	++	–
Thalamus	++	++	+++
Basal ganglia	++	++	+++
Cerebellar cortex	+	++	–

Parameter comparison	Correlation coefficient§
Inflammation vs. EEE virus	0.82
Inflammation vs. lesion on MRI	0.68
EEE virus vs. lesion on MRI	0.52

*Lesions were observed by use of MRI (magnetic resonance imaging). EEE, eastern equine encephalitis; –, absent or none found. †The degree of cerebral inflammation and amount of EEE virus antigen were scored 0–3 on the basis of histopathologic and immunohistopathologic findings. ‡Lesions seen on MRIs were scored 0–3 on the basis of the degree of T2 fluid-attenuated inversion recovery enhancement. §Determined by using the Pearson correlation.

### Treatment

All patients received empiric antimicrobial therapy, and most received acyclovir for empiric coverage of herpes simplex encephalitis. Patients also received antiepileptic medications; immunomodulatory agents, including intravenous immunoglobulin, steroids, cyclophosphamide, and interferon-α2b; and treatments for increased intracranial pressure. Intracranial pressure was monitored for 3 patients (Table 1). No patient underwent intraventricular drain placement or a decompressive craniectomy.

### Characterization of Prognostic Factors

To identify variables associated with clinical outcomes, we compared clinical, laboratory, and imaging findings for patients with favorable outcomes with those for patients with unfavorable outcomes. The length of prodrome was associated with clinical outcome at the time of discharge (Figure 2). The prodrome for patients with a favorable outcome was significantly longer than the prodrome for patients with an unfavorable outcome (median 5 vs. 2 days; p = 0.002). The 8 patients for whom the prodrome lasted <2 days had a poor outcome; all 4 deaths occurred in this group. For these 8 patients, the relative risk was 6.0 for an unfavorable outcome. Of the 7 patients for whom the prodrome lasted >3 days, 6 had a favorable outcome, including 4 patients who fully recovered.

**Figure 2:** Association of length of prodrome with clinical outcome in children with eastern equine encephalitis. Clinical outcome at the time of hospital discharge was defined by using a modified Pediatric Cerebral Performance Category scale (PCPC) (27). Error bars represent standard deviation; the central lines are the mean. The Mann-Whitney rank-sum test was used to compare the prodrome lengths for patients with favorable outcomes with those for patients with unfavorable outcomes. Prodrome is defined as the time from initial illness symptom to first neurologic symptom. Unfavorable outcome is defined as a PCPC score of 4–6, which includes death and severe neurologic disabilities. Favorable outcome is defined as a PCPC score of 1–3, which includes complete recovery and mild to moderate neurologic disability.

Outcome was not associated with any other variable, including patient age, seizure, headache, photophobia, quantity of cerebrospinal fluid pleocytosis, total protein in the cerebrospinal fluid, serum sodium level, or leukocyte count in the blood. Moreover, no significant association was observed between the patient’s clinical outcome and the therapy received.

### Patient Follow-up

Follow-up information was available for 10 of the 11 surviving patients. Duration of follow-up ranged from 3 months to 20 years (median 14.5 months). Patient outcomes at follow-up visits were improved (40%) or unchanged (60%) since the time of discharge. Of the 4 patients with severe deficits at hospital discharge, 3 showed improvement at the follow-up visit (Table 1). Patient 3 progressed from being comatose to being able to communicate nonverbally by 30 months after hospital discharge. Patient 4 recovered speech, comprehension, and the ability to self-feed by 18 months after hospital discharge. Patient 6 progressed from being nonvocal and having limited spontaneous movements at discharge to speaking in full sentences by 21 months after hospital discharge. These results demonstrate the opportunity for substantial cognitive and motor rehabilitation in patients with EEE.

### Pathologic Findings

Tissue sections from the postmortem brain specimens of patients 10 and 12 demonstrated severe meningoencephalitis. Gross findings were notable for marked, diffuse cerebral edema. There was prominent acute and chronic perivascular inflammation within the cortex, thalamus, basal ganglia, and brainstem (Figure 3, panels A, B).

**Figure 3:** Histopathologic features for patient 12 in a study of children with eastern equine encephalitis (EEE), Massachusetts and New Hampshire, 1970–2010. The postmortem samples of central nervous system tissue were obtained 10 days after the onset of symptoms. A) Hematoxylin and eosin (H&E)–stained section of temporal lobe, showing meningeal inflammation (arrow) (magnification ×200). B) H&E-stained section of midbrain, showing perivascular inflammation (arrow) (magnification ×400). C–F) EEE virus (EEEV) colocalizes with areas of tissue injury in the brain. C) H&E-stained section of the basal ganglia, demonstrating foci of marked tissue rarefaction (arrow) (magnification ×12.5). D) Immunohistochemistry of section adjacent to that shown in panel C; staining of the basal ganglia with EEE immune ascites demonstrates foci of EEEV (arrow) that correspond with areas of tissue rarefaction in panel C (magnification ×12.5). E) H&E-stained section of thalamus (magnification ×100). Specificity for EEEV immunoreactivity of this ascites fluid was confirmed by the lack of staining on control brain specimens (data not shown).

A multifocal, patchy distribution of EEEV-stained tissue was observed in specimens from patients10 and 12. In the cerebral hemispheres, clusters of infected cells were predominantly in the gray matter. There were distinct, punched-out islands of pallor in the thalamus and the basal ganglia, representing areas of tissue rarefaction and damage (Figure 3, panels C, E). These regions corresponded to areas demonstrating EEEV infection (Figure 3, panels D, F and Table 5). On the basis of cell morphologic appearance and immunohistochemical staining for neuronal cell marker NeuN, neurons appeared to be the predominant cell population infected by EEEV (Figure 4, panels A–C).

**Figure 4:** Eastern equine encephalitis virus (EEEV) colocalized with neurons in patient 12 in a study of children with eastern equine encephalitis (EEE), Massachusetts and New Hampshire, 1970–2010. A) Immunohistochemistry, using EEEV immune ascites, of the entorhinal temporal cortex, demonstrating EEEV-infected neurons (arrow) (magnification ×400). B) Dual immunohistochemistry with EEE immune ascites (red stain) and a mouse monoclonal anti–neuronal nuclei (NeuN) antibody (brown stain) demonstrates that EEE-infected cells are NeuN–expressing neurons (arrows) (magnification ×400). C) Dual immunohistochemistry with EEE immune ascites (red stain) and rabbit polyclonal anti-glial fibrillary acidic protein (GFAP) antibody (brown stain) demonstrated that EEE-infected cells (arrow) do not express GFAP and are therefore not glial cells (arrowhead) (magnification ×400). Single antibody immunohistochemistry was performed with heat-induced antigen retrieval, using pressure cooker treatment (120°C for 30 s at 15 psi) with citrate buffer, pH 6.0. Lyophilized ascites from American Type Culture Collection (Manassas, VA, USA) was resuspended in 1 mL of double-distilled H_2_0 and stored as stock solutions at −20°C. The ascites stocks were applied at 1:500 for 40 min at room temperature, after which labeled horseradish peroxidase (HRP) anti-mouse antibody was added, and the stocks were incubated 30 min at room temperature. Visualization was performed by using 3,3′-diaminobenzidine chromogen (Dako EnVision+ System-HRP (DAB); Dakocytomation, Carpinteria, CA, USA). Dual immunohistochemistry was performed by using polyclonal rabbit antibody to GFAP (DAKO, Carpinteria CA, USA) at 1:20,000 and mouse monoclonal anti-NeuN, clone A60, MAB377 (Millipore, Billerica, MA, USA) at 1:7,500. Both antibodies were visualized by using the Dako EnVision+ System-HRP (DAB)-brown, and then EEE immune ascites (1:500) was applied, using the alkaline phosphatase method, and visualized again by using Permanent Red (Dako).

The anatomic location of EEEV, inflammatory infiltrates, neuronal death, and rarefied tissue corresponded to the location of the abnormalities seen on MRI (Table 5). These findings provide direct evidence that the abnormalities seen on neuroimaging represent virus-induced inflammatory changes.

## Discussion

Similar to patients described in the original reports of EEE in the United States (12,17), the patients in our study sought care for fever and signs of encephalitis during the late summer months in the New England region of the United States; the patients exhibited a peripheral and CNS neutrophilic immune response. Shortly after seeking care, most patients showed a decline in the level of consciousness and often became comatose. In some patients, encephalitis worsened and was associated with increased intracranial pressure and severe neurologic damage or death. Other patients recovered with minimal or no disability.

The correlation between the length of prodrome and clinical outcome is a key finding of our study. A short prodrome was associated with death or severe neurologic disability; a long prodrome was associated with mild to moderate disability or full recovery. The association between prodrome length and outcome may help identify a subgroup of patients at higher risk for severe disease and for whom more aggressive medical and surgical management may be warranted. In our series, multiple patients showed clinical improvement after medical management of increased intracranial pressure.

Only 3 (20%) patients in our study received intracranial pressure monitoring and none underwent decompressive craniectomy. The treatment choice reflects, in part, the primary signs and symptoms observed at the bedside (intractable seizures vs. signs of uncontrolled cerebral swelling) and changes in medical practice over the past 4 decades. Neuroimaging conducted early in the course of the disease disclosed scattered, focal lesions without gross swelling or herniation; however, severe swelling was clearly present at autopsy. Barnett and colleagues (18) observed severe cerebral swelling, peaking around day 12, in 60% of adults with encephalitis. Furthermore, in that early series, all patients with low intracranial pressure (<20 mm Hg) survived, and nearly all those with increased intracranial pressure died. More recently, routine intracranial pressure monitoring and cerebral perfusion pressure–directed therapies have been recommended for patients with worsening encephalitis (19). Decompressive craniectomy has been suggested for patients with conditions refractory to medical management (20,21).

We did not observe some previously reported associations between poor outcome and the degree of CSF pleocytosis (3), serum hyponatremia (3), age (4), or electroencephalogram abnormalities (4). The small sample size in our study may explain the difference between findings in our study and others; power calculations indicate that our case series would not be expected to show these associations.

A limitation of our study is the potential for recall bias with regard to timing of the prodrome. The timing and symptoms of the prodrome in this study depended on the recall of the patient (or parent) and documentation by the medical provider. However, these are the same data that clinicians use to make treatment decisions, so this association, despite recall bias, is probably clinically relevant. Prospective study of EEE with larger sample sizes would overcome some of the limitations of this study and would be a means of assessing the utility of prodrome length as a clinically relevant tool for predicting outcome. However, the rarity of the disease makes a prospective study difficult to perform.

Our finding of the predilection for focal basal ganglia lesions and thalamic lesions in patients with EEEV is consistent with findings in prior studies (3,22–24). In addition to the characteristic basal ganglia and thalamic lesions, we observed a pattern of focal cortical lesions in almost all patients. This finding extends the neuroimaging findings of EEE to include focal cortical lesions in children. The prominence of cortical lesions in EEE in children may reflect pathophysiologic differences between children and adult patients. It remains unclear why EEE in children causes a characteristic pattern of multifocal lesions with a predilection for the basal ganglia, thalamus, and cortex. Other viral encephalitides also exhibit a tropism for certain neuroanatomic locations. For example, Japanese encephalitis often affects the thalamus and basal ganglia (25), and herpes simplex virus has a predilection for the temporal lobes. Recognition that focal cortical CNS lesions are characteristic features of EEE in children may assist in the diagnosis of EEE.

In addition, in our study, the rate of seizures (>70%), specifically, complex partial seizures, was higher than that found by Deresiewicz et al. (25%) (3). Because complex partial seizures are often secondary to focal cortical lesions, it is tempting to speculate that these focal cortical lesions caused more complex partial seizures.

The pattern of histologic findings in our study, including inflammation of the leptomeninges; chronic and acute perivascular inflammation; evidence of neuronal injury and cell death (11,26,27); and presence of EEEV antigen specifically in neurons, is similar to the pattern of findings in previous reports (27). We extend these observations by demonstrating that lesions observed on radiographs correspond to pathologic regions of EEEV infection and CNS inflammation.

## Conclusions

The rates of illness and death are high among children with EEE. Key features of this disease include signs of encephalitis during late summer and early fall, neutrophilic cerebrospinal fluid pleocytosis, and abnormal neuroimaging results, including the finding of lesions in the basal ganglia and cerebral cortex. A short prodrome is associated with unfavorable outcomes and, when present, warrants close monitoring and management of intracranial pressure.

## References

[1] Centers for Disease Control and Prevention. Eastern equine encephalitis—New Hampshire and Massachusetts, August–September 2005. MMWR Morb Mortal Wkly Rep. 2006;55:697–700 .16810146

[2] Calisher CH. Medically important arboviruses of the United States and Canada. Clin Microbiol Rev. 1994;7:89–116 .811879210.1128/cmr.7.1.89PMC358307

[3] Deresiewicz RL, Thaler SJ, Hsu L, Zamani AA. Clinical and neuroradiographic manifestations of eastern equine encephalitis. N Engl J Med. 1997;336:1867–74. 10.1056/NEJM1997062633626049197215

[4] Przelomski MM, O'Rourke E, Grady GF, Berardi VP, Markley HG. Eastern equine encephalitis in Massachusetts: a report of 16 cases, 1970–1984. Neurology. 1988;38:736–9. 10.1212/WNL.38.5.7363362371

[5] Centers for Disease Control and Prevention. Eastern equine encephalitis: epidemiology and geographic distribution [cited 2012 Sept 25]. http://www.cdc.gov/EasternEquineEncephalitis/tech/epi.html#moremapschartstables

[6] Eastern equine encephalitis (EEE). Department of Health: information for a healthy New York [cited 2012 Oct 1]. http://www.health.ny.gov/diseases/communicable/eastern_equine_encephalitis/fact_sheet.htm

[7] Health and Human Services. Massachusetts. Massachusetts arbovirus surveillance: humans [cited 2012 Sept 25]. http://westnile.ashtonweb.com/index42.asp

[8] Vermont.gov—the official state website. News releases. Health commissioner confirms second death from EEE [cited 2012 Sept 25]. http://www.vermont.gov/portal/government/article.php?news=3834

[9] Gibney KB, Robinson S, Mutebi JP, Hoenig DE, Bernier BJ, Webber L, Eastern equine encephalitis: an emerging arboviral disease threat, Maine. Vector Borne Zoonotic Dis. 2011;11:637–9. 10.1089/vbz.2010.018921254938

[10] Eastern equine encephalitis—Canada (03) (Nova Scotia). ProMed. 2009 Nov 3. http://www.promedmail.org, archive no. 20091105.3826.

[11] Feemster RF. Outbreak of encephalitis in man due to the eastern virus of equine encephalomyelitis. Am J Public Health Nations Health. 1938;28:1403–10. 10.2105/AJPH.28.12.140318014957PMC1527806

[12] Farber S, Hill A, Connerly ML, Dingle JH. Encephalitis in infants and children caused by the virus of the eastern variety of equine encephalitis. JAMA. 1940;114:1725–31. 10.1001/jama.1940.02810180001001

[13] Letson GW, Bailey RE, Pearson J, Tsai TF. Eastern equine encephalitis (EEE): a description of the 1989 outbreak, recent epidemiologic trends, and the association of rainfall with EEE occurrence. Am J Trop Med Hyg. 1993;49:677–85 .827963510.4269/ajtmh.1993.49.677

[14] Centers for Disease Control and Prevention. Arboviral diseases, neuroinvasive and non-neuroinvasive; 2011 case definition [cited 2011 Sept 28]. http://wwwn.cdc.gov/NNDSS/beta/bcasedef.aspx?CondYrID=616&DatePub=1/1/2011 12:00:00 AM

[15] Levitt LP, Lovejoy FH Jr, Daniels JB. Eastern equine encephalitis in Massachusetts—first human case in 14 years. N Engl J Med. 1971;284:540. 10.1056/NEJM1971031128410085542572

[16] Fiser DH. Assessing the outcome of pediatric intensive care. J Pediatr. 1992;121:68–74. 10.1016/S0022-3476(05)82544-21625096

[17] Fothergill LD, Dingle JH, Farber S, Connerly ML. Human encephalitis caused by the virus of the eastern variety of equine encephalomyelitis. N Engl J Med. 1938;219:411. 10.1056/NEJM193809222191201

[18] Barnett GH, Ropper AH, Romeo J. Intracranial pressure and outcome in adult encephalitis. J Neurosurg. 1988;68:585–8. 10.3171/jns.1988.68.4.05853351587

[19] Shetty R, Singhi S, Singhi P, Jayashree M. Cerebral perfusion pressure—targeted approach in children with central nervous system infections and raised intracranial pressure: is it feasible? J Child Neurol. 2008;23:192–8. 10.1177/088307380730871618263756

[20] Di Rienzo A, Iacoangeli M, Rychlicki F, Veccia S, Scerrati M. Decompressive craniectomy for medically refractory intracranial hypertension due to meningoencephalitis: report of three patients. Acta Neurochir (Wien). 2008;150:1057–65. 10.1007/s00701-008-0019-118773140

[21] González Rabelino GA, Fons C, Rey A, Roussos I, Campistol J. Craniectomy in herpetic encephalitis. Pediatr Neurol. 2008;39:201–3. 10.1016/j.pediatrneurol.2008.06.00618725067

[22] Golomb MR, Durand ML, Schaefer PW, McDonald CT, Maia M, Schwamm LH. A case of immunotherapy-responsive eastern equine encephalitis with diffusion-weighted imaging. Neurology. 2001;56:420–1. 10.1212/WNL.56.3.42011171919

[23] Lury KM, Castillo M. Eastern equine encephalitis: CT and MRI findings in one case. Emerg Radiol. 2004;11:46–8. 10.1007/s10140-004-0350-715309665

[24] Morse RP, Bennish ML, Darras BT. Eastern equine encephalitis presenting with a focal brain lesion. Pediatr Neurol. 1992;8:473–5. 10.1016/0887-8994(92)90013-O1476580

[25] Kalita J, Misra UK. Comparison of CT scan and MRI findings in the diagnosis of Japanese encephalitis. J Neurol Sci. 2000;174:3–8. 10.1016/S0022-510X(99)00318-410704974

[26] Kim JH, Booss J, Manuelidis EE, Duncan CC. Human eastern equine encephalitis. Electron microscopic study of a brain biopsy. Am J Clin Pathol. 1985;84:223–7 .402522910.1093/ajcp/84.2.223

[27] Garen PD, Tsai TF, Powers JM. Human eastern equine encephalitis: immunohistochemistry and ultrastructure. Mod Pathol. 1999;12:646–52 .10392642

# Review article: Eastern Equine Encephalitis in Children, Massachusetts and New Hampshire,USA, 1970–2010

**Authors:** Michael A. Silverman, John Misasi, Sandra Smole, Henry A. Feldman, Adam B. Cohen, Sandro Santagata, Michael McManus, Asim A. Ahmed

**Affiliations:** Author affiliations: Children's Hospital Boston, Boston, Massachusetts, USA (M.A. Silverman, J. Misasi, H.A. Feldman, S. Santagata, M. McManus, A.A. Ahmed);; Department of Public Health, Boston (S. Smole); Massachusetts General Hospital, Boston (A.B. Cohen);; Brigham and Women’s Hospital, Boston (S. Santagata)

## Medscape CME Quiz

## Earning CME Credit

To obtain credit, you should first read the journal article. After reading the article, you should be able to answer the following, related, multiple-choice questions. To complete the questions (with a minimum 70% passing score) and earn continuing medical education (CME) credit, please go to www.medscape.org/journal/eid. Credit cannot be obtained for tests completed on paper, although you may use the worksheet below to keep a record of your answers. You must be a registered user on Medscape.org. If you are not registered on Medscape.org, please click on the New Users: Free Registration link on the left hand side of the website to register. Only one answer is correct for each question. Once you successfully answer all post-test questions you will be able to view and/or print your certificate. For questions regarding the content of this activity, contact the accredited provider, CME@medscape.net. For technical assistance, contact CME@webmd.net. American Medical Association’s Physician’s Recognition Award (AMA PRA) credits are accepted in the US as evidence of participation in CME activities. For further information on this award, please refer to http://www.ama-assn.org/ama/pub/category/2922.html. The AMA has determined that physicians not licensed in the US who participate in this CME activity are eligible for *AMA PRA Category 1 Credits*™. Through agreements that the AMA has made with agencies in some countries, AMA PRA credit may be acceptable as evidence of participation in CME activities. If you are not licensed in the US, please complete the questions online, print the certificate and present it to your national medical association for review.

## Article Title:  Eastern Equine Encephalitis in Children, Massachusetts and New Hampshire, USA, 1970–2010

## CME Questions

**1. You are evaluating a 10-year-old girl with a 2-day history of fever and malaise. The girl’s parents are worried because of reports of 2 confirmed cases of Eastern equine encephalitis (EEE) in the state during the past 3 weeks. Based on the current study by Silverman and colleagues, which of the following statements regarding the clinical presentation of EEE is most accurate?**

A. Cases were diagnosed between November and February

B. Half of patients had fever at the time of presentation for medical care

C. All patients had symptoms or signs of meningoencephalitis at the time of presentation for medical care

D. The mean age of patients was approximately 10 years

**2. What should you consider regarding findings on ancillary studies of children with EEE in the current study?**

A. Neutrophilic cerebrospinal fluid (CSF) pleocytosis was present in all patients

B. CSF protein levels were generally normal

C. Epileptiform activity was universal among patients who received EEG

D. Lesions were most common in the pons on neuroimaging studies

**3. The patient is diagnosed with EEE. Which of the following variables was most important in predicting a worse outcome in the current study?**

A. Younger age

B. Seizure at the time of presentation

C. Higher levels of total protein in CSF

D. Shorter prodrome

**4. The patient’s parents are very concerned regarding the prognosis for their daughter. What was the most common outcome of EEE in the current study?**

A. Death or severe neurologic deficits

B. Some form of mild neurologic impairment only

C. Seizure disorder only

D. Complete recovery with no sequelae

## Activity Evaluation

**Table:** 

**1. The activity supported the learning objectives.**				
Strongly Disagree				Strongly Agree
1	2	3	4	5
**2. The material was organized clearly for learning to occur.**				
Strongly Disagree				Strongly Agree
1	2	3	4	5
**3. The content learned from this activity will impact my practice.**				
Strongly Disagree				Strongly Agree
1	2	3	4	5
**4. The activity was presented objectively and free of commercial bias.**				
Strongly Disagree				Strongly Agree
1	2	3	4	5
